# Winners and losers: tropical forest tree seedling survival across a West African forest–savanna transition

**DOI:** 10.1002/ece3.2133

**Published:** 2016-04-18

**Authors:** Anabelle W. Cardoso, José A. Medina‐Vega, Yadvinder Malhi, Stephen Adu‐Bredu, George K.D. Ametsitsi, Gloria Djagbletey, Frank van Langevelde, Elmar Veenendaal, Immaculada Oliveras

**Affiliations:** ^1^ Environmental Change InstituteSchool of Geography and the EnvironmentUniversity of OxfordOxfordUK; ^2^ Plant Ecology and Nature Conservation GroupWageningen UniversityWageningenThe Netherlands; ^3^ Resource Ecology GroupWageningen UniversityWageningenThe Netherlands; ^4^ Forestry Research Institute of Ghana (FORIG) of the Council for Scientific and Industrial Research (CSIR)KumasiGhana

**Keywords:** Drought, fire, forest encroachment, functional traits

## Abstract

Forest encroachment into savanna is occurring at an unprecedented rate across tropical Africa, leading to a loss of valuable savanna habitat. One of the first stages of forest encroachment is the establishment of tree seedlings at the forest–savanna transition. This study examines the demographic bottleneck in the seedlings of five species of tropical forest pioneer trees in a forest–savanna transition zone in West Africa. Five species of tropical pioneer forest tree seedlings were planted in savanna, mixed/transition, and forest vegetation types and grown for 12 months, during which time fire occurred in the area. We examined seedling survival rates, height, and stem diameter before and after fire; and seedling biomass and starch allocation patterns after fire. Seedling survival rates were significantly affected by fire, drought, and vegetation type. Seedlings that preferentially allocated more resources to increasing root and leaf starch (starch storage helps recovery from fire) survived better in savanna environments (frequently burnt), while seedlings that allocated more resources to growth and resource‐capture traits (height, the number of leaves, stem diameter, specific leaf area, specific root length, root‐to‐shoot ratio) survived better in mixed/transition and forest environments. Larger (taller with a greater stem diameter) seedlings survived burning better than smaller seedlings. However, larger seedlings survived better than smaller ones even in the absence of fire. *Bombax buonopozense* was the forest species that survived best in the savanna environment, likely as a result of increased access to light allowing greater investment in belowground starch storage capacity and therefore a greater ability to cope with fire. *Synthesis*: Forest pioneer tree species survived best through fire and drought in the savanna compared to the other two vegetation types. This was likely a result of the open‐canopied savanna providing greater access to light, thereby releasing seedlings from light limitation and enabling them to make and store more starch. Fire can be used as a management tool for controlling forest encroachment into savanna as it significantly affects seedling survival. However, if rainfall increases as a result of global change factors, encroachment may be more difficult to control as seedling survival ostensibly increases when the pressure of drought is lifted. We propose *B. buonopozense* as an indicator species for forest encroachment into savanna in West African forest–savanna transitions.

## Introduction

Tropical forests are generally defined as a continuous closed‐canopy tree layer with a discontinuous herbaceous understory. By contrast, tropical savannas have a discontinuous tree layer which lies within a continuous herbaceous layer (Torello‐Raventos et al. 2013; Lehmann et al. 2014; Veenendaal et al. 2014). Tropical humid forests are largely unaffected by regular fires (Cochrane et al. 1999; Hoffmann et al. 2012), while drier forests are affected only by occasional ground fires (Bond and van Wilgen 1996). Tropical savannas, however, as a result of their continuous herbaceous fuel layer, are one of the most fire‐prone ecosystems in the world (Mouillot and Field 2005), with 2.6 million km^2^ of savanna burning in Africa each year (Schultz et al. 2008).

Although the balance of forest and savanna is dynamic through time and space, the loss of savanna as a result of forest encroachment has been occurring at an unprecedented rate in the recent years. Forest encroachment has already been reported in Southern (Parr et al. 2012), Western (Fairhead and Leach 1996), Eastern (Belsky and Amundson 1992), and Central Africa (Mitchard et al. 2009). In some sites, it is proceeding as rapidly as 50 m per century (Schwartz et al. 1996), and in fire‐protected areas, there can be a complete transformation from savanna to forest in just 15 years (Jeffery et al. 2014). The loss of savanna habitat as a result of forest encroachment is concerning as savannas are “globally extensive, provide critical ecosystem services, and influence the earth‐atmosphere system [providing] significant environmental, economic, and cultural value to the world” (Parr et al. 2014).

Fire is a key factor affecting the degree of forest encroachment that occurs as it is a key driver in maintaining the open canopy of savannas by reducing tree cover (Higgins et al. 2007). As a result of this, fire has the potential to determine whether forest or savanna exists at a point in space in time. Fires in savanna typically burn the herbaceous layer, with trees and saplings not tall enough to escape the flames experiencing top kill (whole or partial death of aboveground biomass) (Trollope 1984; Glitzenstein et al. 1995; Williams et al. 1999; Higgins et al. 2012). Top kill in savannas seldom leads to plant death (Bond and van Wilgen 1996; Hoffmann et al. 2009), and repeated top kill by fire, when it does not lead to plant death, leads to a demographic bottleneck with a large number of trees and saplings unable to reach canopy height (Bond and van Wilgen 1996; Higgins et al. 2000, 2012; Bond and Keeley 2005). Many forest–savanna transitions are experiencing a shift in the fire regime toward decreased fire recurrence as a result of, *inter alia*, fire extinction policies, rural area abandonment, and changes in traditional cultural and spiritual practices. These changes in fire regime have been associated with increased expansion of forest tree species into savanna areas (Mitchard et al. 2009; Geiger et al. 2011; Mitchard and Flintrop 2013). In addition to these local drivers, global drivers, specifically increasing atmospheric CO_2_, have also been hypothesized to be exacerbating the expansion of forests into savannas. Historically, some of the largest scale changes in vegetation cover have been associated with changes in atmospheric CO_2_, which enhances the growth of trees over grasses (Ogren 1984; Dai et al. 1993; Kgope et al. 2010). In greenhouse experiments, African *Acacia* seedlings showed increased photosynthesis, total stem, total stem diameter, shoot dry weight, and root dry weight under increased CO_2_ concentrations, implying that CO_2_ has a direct effect on these seedlings' ability to recruit in savanna systems (Kgope et al. 2010).

The process of forest encroachment into savanna is especially relevant in West Africa where our study takes place. According to the global circulation models, under climate change predictions alone the savannas of West Africa are predicted to contract by 2050, with tree cover predicted to increase by 1–10% in large parts of Benin, Burkina Faso, Côte D'Ivoire, Ghana, and Togo (Heubes et al. 2011). In many places in West Africa, protected areas were established in the early 1960's with the aim of maintaining a high forest cover in order to protect natural water resources (Hagan 1998), among other reasons. These reserves, especially in Ghana, were largely concentrated in the transitional areas between the Guinea savanna and dry semideciduous forest, and one of the most important implications of this was the resulting severe fire suppression policies that altered the fire regime of these areas.

Forest encroachment into savannas is thought to be the result of establishment of forest tree species rather than savanna tree species (Geiger et al. 2011), with the first step of encroachment being the establishment of pioneer forest tree seedlings in the forest–savanna transition zone. Demographic bottlenecks play a large role in determining tree densities in savannas (Higgins et al. 2007; Staver et al. 2011), and the establishment phase of forest tree seedlings represents one of these demographic bottlenecks. This bottleneck is thought to be primarily caused by competitive exclusion of grasses, soil fertility (Veenendaal et al. 1996a; Viani et al. 2011), water availability (Veenendaal et al. 1996b), and fire vulnerability (Hoffmann et al. 2012). Seedling strategies to survive this establishment bottleneck will therefore affect the degree of forest encroachment that occurs. This study investigates the survival of five common light‐demanding forest tree seedlings across a forest–savanna transition zone. Specifically, it examines the environmental factors and seedling traits that may be affecting seedling survival patterns during this establishment phase. The hypotheses of this study were as follows: (1) Seedlings will show differential survival patterns between vegetation types (forest, mixed/transition, savanna); (2) seedlings that are larger (greater height and diameter) before fire are more likely to survive burning; and (3) differential survival across vegetation types is associated with distinct resource‐capture and growth trait features.

## Materials and Methods

### Study site and experimental design

The study took place in Kogyae Strict Nature Reserve (SNR), Ghana, West Africa (1°05′W, 7°15′N). Kogyae SNR is 386 km^2^, and altitude ranges from 120 to 215 m asl. Kogyae SNR is situated in the northeastern part of the Ashanti region of Ghana. Kogyae SNR lies on the border between Guinea–Congolian rainforest of the drier type and a mosaic of lowland rainforest and secondary grassland (White 1983), with the large majority of its area falling into the latter. Kogyae SNR is the largest protected area in Ghana that covers the transitional vegetation zone between savanna and forest, making it an ideal study site for this experiment. Grazing intensity in the study area is negligible, and there is no active fire management in the Reserve. However, escaping fires from nearby human activities occur at a high frequency (almost annually) in the southern area where the plots were located, burning the savanna and mixed areas and, less frequently, the forest areas (Janssen et al. in review).

Mean annual rainfall at Kogyae SNR is 1453 mm/year (Domingues et al. 2010), and there are two rainy seasons between May and October, with precipitation peaking in June and September. The geology of the KSNR belongs to the Voltarian system, and the rocks are reddish brown sandstone whenever exposed. The soil in the savanna and transition areas is Haplic Arenosols (Dystric), and the soil of the forest sites Haplic Nitosols (Dystric) (Domingues et al. 2010). Nutrient analysis showed soils across the study area to be strongly nitrogen limited; however, there were no relevant differences in soil nutrients between vegetation types (S. Moore, unpubl. data).

Twenty‐four 10 m × 10 m plots were randomly set up in Kogyae SNR with eight plots in each of the three vegetation types: savanna, transition zone (or mixed vegetation), and forest, following Torello‐Raventos et al. (2013). Five common tropical West African forest tree species were chosen (Table 1): *Nauclea didderrichii* (De Wild. & T.Durand) Merrill (representing moist forest species)*, Khaya ivorensis* A. Chev.*, Triplochiton scleroxylon* K. Schum.*, Terminalia superba* Engl. & Diels (climatically ubiquitous species across the forest zone with *T. superba* occurring in dry semideciduous forest), and *Bombax buonopozense* P. Beauv. (species occurring in the driest forest). Of these, only *B. buonopenze* has been recorded to occur in Kogyae, although Kogyae falls into the distribution range of all species (Hawthorne and Jongkind 2006). All of these species have dispersal times in February–March and reproduce by seeds. Forty seedlings, eight seedlings of each of the five study species, were randomly and equidistantly (2 × 1.25 from each other) planted in each plot, for total of 960 seedlings in the study. Seedlings were grown from seed in identical nursery conditions for three months prior to the beginning of the experiment before being planted in the rainy season. When seedlings were planted, all of the aboveground herbaceous cover was removed in order to minimize aboveground competition during the seedlings' establishment phase. Seedlings were planted in June 2013, and the study ran over the course of a year until June 2014, when all surviving seedlings were uprooted. Fire burnt the majority of the plots in February 2014.

**Table 1:** Ecological characteristics of the five selected species for the study

Species	Family	Guild	Distribution^1^	Rainfall range^2^ (mm)
*Bombax buonopezense*	Bombaceae	Pioneer	Dry forest	800–1500
*Khaya ivorensis*	Meliaceae	Nonpioneer light demander	Moist–dry forest	1250–3000
*Nauclea diderrichii*	Rubiacaeae	Pioneer	Wet–moist forest	1600–3000
*Terminalia superba*	Combretaceae	Pioneer	Moist–dry forest	1000–1800
*Triplochiton scleroxylon*	Sterculiacea	Pioneer	Moist–dry forest	1000–3000

Sources: ^1^Hawthorne (1995), ^2^Kindt et al. (2015).

### Seedling's trait data

At each plot, height, basal stem diameter, and the number of leaves of each surviving seedling were measured in five censuses, three before the fire (June and September 2013 and January 2014) and two after the fire (April and June 2014). The number of surviving seedlings was recorded at each time step. All surviving seedlings were uprooted in June 2014, and root length was measured. After uprooting, all leaves on each seedling were scanned, and total leaf area of each seedling was calculated using an already existing code in MATLAB (code is publically available on GitHub https://github.com/bblonder/leafarea) and described by Neyret et al. (in review). All materials were oven‐dried at 72°C until constant weight to obtain root, stem, and leaf dry biomass.

Root, leaf, and stem materials were analyzed for starch content following the protocol of Duranceau et al. (1999) and Damesin et al. (2013). The 91 seedlings that survived until the end of the study period were used for the starch analysis. Each seedling was cut into three parts (roots, stems, and leaves), and each of these samples was classified according to species, vegetation type (forest, savanna, and mixed), and fire occurrence (burnt and unburnt). Where enough material was available, all composite samples were analyzed in triplicate. However, some samples were too small to be analyzed for starch concentration. In total, 159 starch concentration analyses were performed.

### Environmental measurements

Herbaceous biomass in each plot was recorded before seedlings were planted and before the fire (November 2013) using a disk pasture meter that was calibrated for each vegetation type following the method described by Dörgeloh (2002) (Figure S1). In January and June 2014, we took 10 height readings in each plot and obtained herbaceous biomass estimates using the averaged value per plot and the calibration equation (Figure S1). Litterfall, being a fine fuel for fire, was collected in January 2014 in 50 cm × 50 cm subplots located adjacent corner of the seedlings plots, therefore in the same vegetation to the seedling plots.

In order to determine the level of canopy openness at each plot, hemispheric pictures were taken in November 2013 (end of rainy season) using a Nikon (R) E4500 camera with a fish‐eye lens on a tripod mounted at 1.50 m at 180° (Marthews et al. 2012). Pictures were underexposed by one stop in order to enhance contrast (Zhang et al. 2005). These pictures were used to calculate canopy openness (%) using the Gap Light Analyser imaging software (Frazer et al. 1999).

Total precipitation was recorded at the closest meteorological station in Ejura (25 km away). Precipitation data were only available from September 2013 to June 2014, with a gap between 17 April and 27 May 2014. The total precipitation for this period was therefore estimated by applying a mean daily precipitation based on the mean daily total precipitation of the 15 preceding and proceeding days of the missing data period (i.e., the mean of 2–16 April 2014 and 28 May–10 June 2014). Mean cumulative water deficit (MCWD) was used as an indicator of water availability.

A fire burnt the area on 2 February 2014, but left a few plots unburnt that were classified as “unburnt” (vs. “burnt” plots). There was one unburnt plot in the savanna, three unburnt plots in the mixed vegetation type, and two unburnt plots in the forest.

### Data analysis

Significant differences in canopy openness, herbaceous biomass, and litterfall among vegetation types were examined using a Kruskal–Wallis test (herbaceous biomass and canopy openness) and a one‐way ANOVA (litterfall).

To see whether larger seedlings had a survival advantage over smaller ones, we conducted analysis taking into account the unbalanced design after fire. Differences in total survival between species and vegetation types before the fire (data from January 2014) and after the fire (data from April and June 2014) were examined using a Mantel–Cox log‐rank test (Bland and Altman 2004). To look for the effect of prefire height and prefire diameter on seedling survival, we did a generalized linear model (GLM) with a binomial distribution (Zuur et al. 2009). Due to low sample sizes, the analysis could not look for species‐specific effects.

Measured traits of all surviving seedlings in January 2014 and in June 2014 were compared between species using the Kruskal–Wallis tests (Sokal and Rohlf 1995). The trait variables assessed were height, diameter, and the number of leaves in January and June 2014, plus leaf dry mass, root dry mass, stem dry mass, specific root length (root length/root dry mass), root‐to‐shoot ratio (root dry mass/[stem + leaf dry mass]), leaf starch concentration, root starch concentration, stem starch concentration, and specific leaf area (leaf area/leaf dry mass) in June 2014.

To examine which factors better explained the surviving proportion of seedlings after one year, taken to be the number of surviving seedlings at each time period divided by the total number of seedlings planted at time zero, we used a generalized linear mixed‐effects model (GLMM) with a binomial distribution (Zuur et al. 2009). Vegetation type, species, and fire were introduced in the model as fixed factors; plot was introduced as a random factor, and cumulative rainfall as a covariate. Time series for each census was expressed through cumulative rainfall. Survival of the different species in the different vegetation types after the fire was further explored with a principle component analysis (PCA) which was carried out on all surviving seedlings' measured traits (Sokal and Rohlf 1995). Differences in PCA scores between plots in different vegetation types were explored using multivariate analysis of variance (MANOVA). Tukey posthoc tests were then performed to detect differences in PCA scores between species and vegetation types.

All analyses were performed in R v3.1.1 (R Core Team 2014) and SPSS v22.0.0.1\armonl, NY: IBM Corp. In all statistical tests, overall differences were noted significant when *P* < 0.05 unless otherwise stated.

## Results

### Seedling survival differs between vegetation types

At the time of harvesting in June 2014, 91 of the original 960 planted tree seedlings were alive, representing 9.5% of the total (Table 2). At the last census, the highest survival rate for seedlings was observed in savanna plots, where 13% of the original seedlings planted survived, followed by the mixed plots with 11% survival and the forest plots with 4% survival (Table 2). The savanna vegetation type had significantly more herbaceous biomass, significantly less litterfall, and a significantly more open canopy than the other two vegetation types (Table S1).

**Table 2:** Percentage of seedling survival before the fire (prefire, Jan 2014) and at the time of harvesting (postfire, June 2014) for each species in each vegetation type. *n* = 320 seedlings for each vegetation type, proportioned equally between species (64 seedlings per species per vegetation type). BB = *B.buonopozense,* KI = *K.ivorensis*, TS =*T.superba* and WW = *T.scleroxylon*. Surv = total survival percentage

Species	Forest	Mixed	Savanna
Prefire (%)			
BB	55	54	88
KI	46	49	70
ND	5	4	29
TS	25	53	45
WW	72	84	39
Surv prefire	40	49	54
Postfire (%)			
BB	5	24	42
KI	3	9	8
ND	0	0	0
TS	2	21	4
WW	12	19	11
Surv postfire	4	14	13

**Figure 1:**
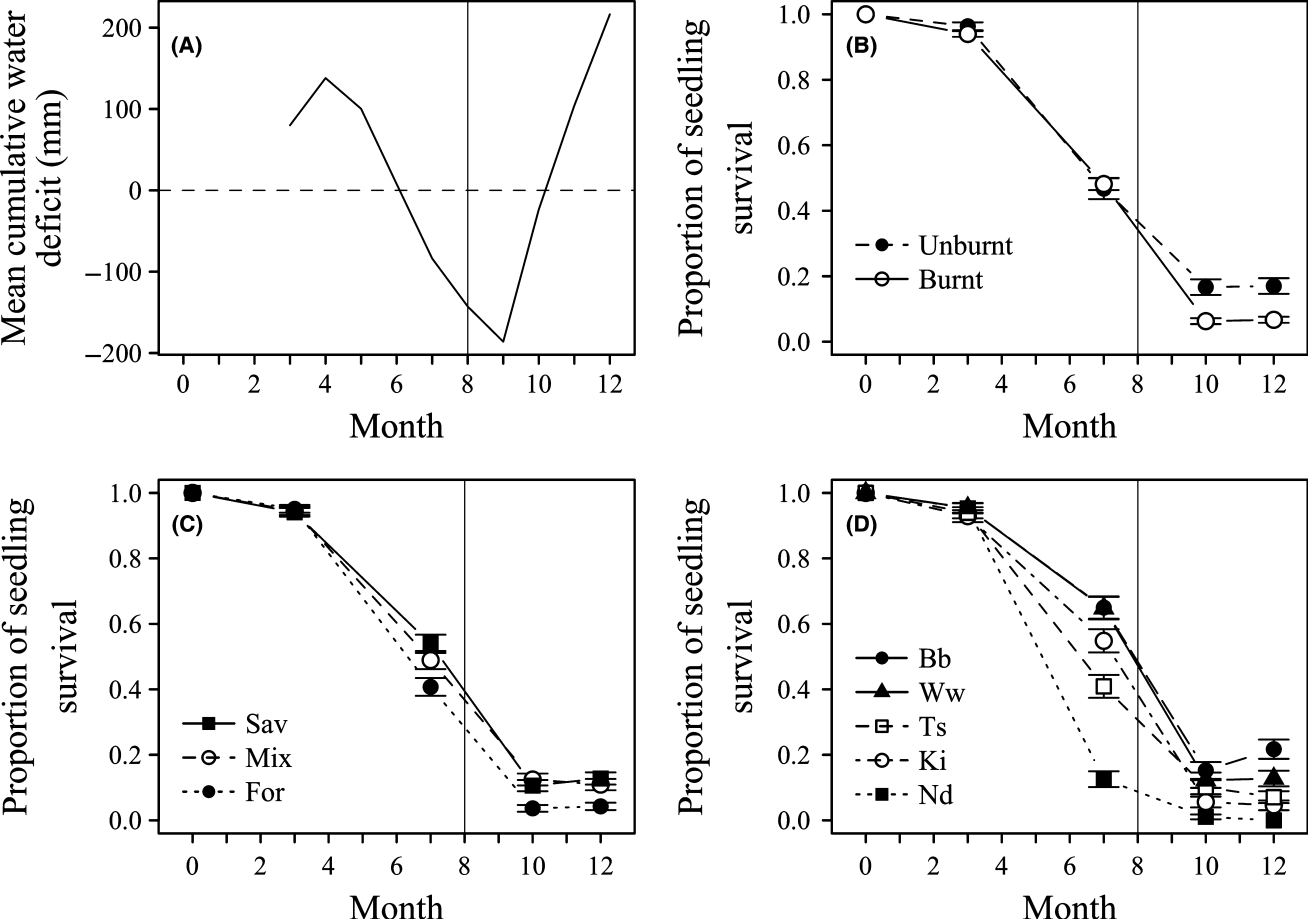
(A) Precipitation over the year from June 2013 (month 0) to June 2014 (month 12). (B) Seedling survival curves over this time period for seedlings in burnt and unburnt plots, (C) seedlings in the different vegetation types, and (D) seedlings of the different species. Species names are as follows: Bb = *B. buonopozense,* Ki = *K. ivorensis*, Nd = *N. diderrichii*, Ts = *T. superba*, and Ww = *T. scleroxylon*. Month number is relative to the month of planting, with planting month = 0 and harvesting month = 12. The vertical back line indicates the time of the fire. Error bars show 95% confidence intervals around the mean.

Before the fire (i.e., in the seedling establishment phase), increased MCWD during the dry season (October–March) was associated with increased seedling mortality, with 50% of seedlings dying (Fig. 1, Table 2). *N. diderrichii* showed the sharpest decrease in survival of all species (64% of mortality, Tables 2 and S2), with survival dropping to zero following the fire (Fig. 1, Table 2). *B. buonopozense* and *T. scleroxylon* had the highest survival rates before the fire (in the establishment phase) (49% and 43% of mortality, respectively).

The GLMM model provided a good fit of the observed data (*F*_30, 3903_ = 70.8, *P* < 0.0001), corroborating that vegetation type (*F*_2, 3903_ = 11.0, *P* < 0.0001), fire (*F*_1, 3903_ = 59.7, *P* < 0.0001), cumulative rainfall (*F*_1, 3903_ = 24.0, *P* < 0.0001), and their interaction (vegetation type × fire *F*_2, 3903_ = 7.2, *P* = 0.001; fire x cumulative rainfall *F*_1, 3903_ = 30.1, *P* = 0.017) influenced seedling's survival. However, the species effect was not significant, showing that there were no species‐specific influences on survival.

### Larger seedlings survived better independent of whether or not they were burnt

**Figure 2:**
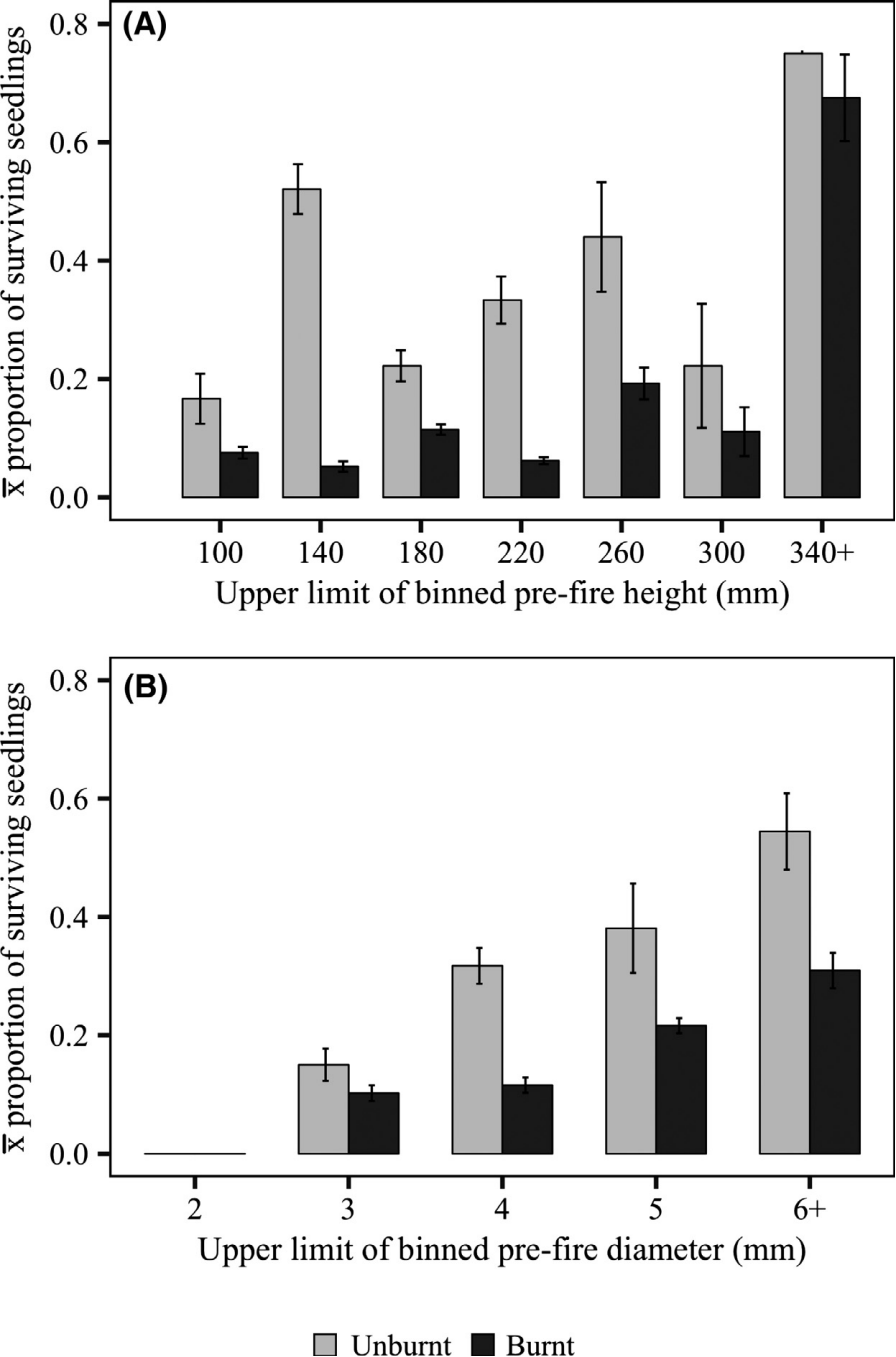
Average proportion of surviving seedlings in each size class (upper limit of bin shown) for height (A) and diameter (B) for burnt and unburnt plots. Error bars show standard error.

Seedling survival after burning was significantly affected by seedling prefire height (Wald *χ* ^2^ = 18.2, df = 6, *P* = 0.006, Fig. 2) and by fire (Wald *χ* ^2^ = 12.2, df = 2, *P* < 0.0001); however, the interaction between height and fire was not significant (*χ* ^2^ = 5.30, df = 6, *P* = 0.540). Similarly, basal diameter before the fire (Wald *χ* ^2^ = 25.1, df = 7, *P* = 0.001) and fire (Wald *χ* ^2^ = 18.9, df = 1, *P* < 0.003) had a significant effect on survival rates, but their interaction did not (Wald *χ* ^2^ = 5.2, df = 7, *P* = 0.520). In other words, seedlings that were larger (greater height and diameter) before the fire had an increased survival rate regardless of whether or not they were burnt, and burning had a negative effect on survival regardless of prefire size (Fig. 2).

### Differential survival across vegetation types is associated with distinct trait features

**Figure 3:**
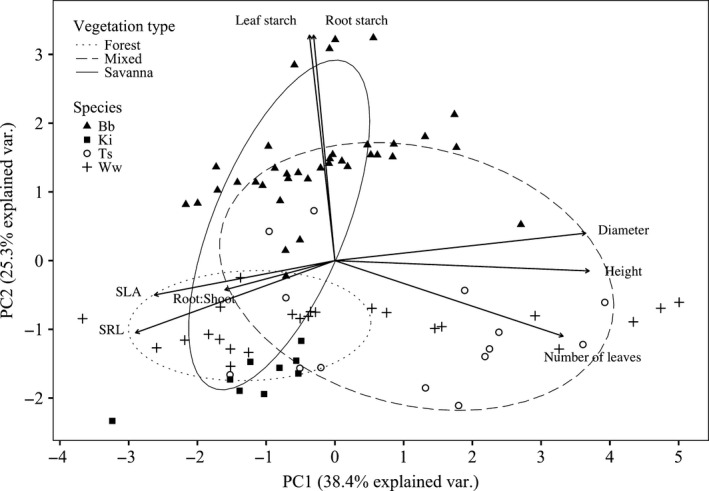
Results of a principle component analysis of all surviving seedlings. Principle components (PC's) 1 and 2 cumulatively explain 63.7% of the variation within the data. Variables included were as follows: leaf starch concentration, twig starch concentration, root starch concentration, root:shoot (g:g), specific leaf area (SLA) (cm^2^/g), specific root length (SRL) (mm/g dry weight), diameter, height, and the number of leaves. Species names are as follows: Bb = *B. buonopozense,* Ki = *K. ivorensis*, Nd = *N. diderrichii*, Ts = *T. superba*, and Ww = *T. scleroxylon*. Ellipses around species were drawn using a 0.68 probability normal distribution.

**Figure 4:**
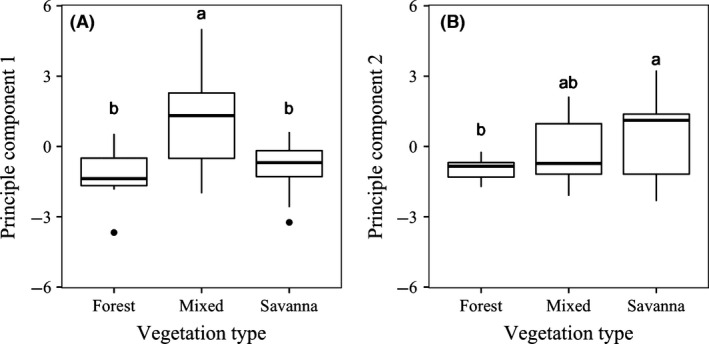
Results of MANOVA that looked for differences in principle component scores (from Fig. 3 analysis) between vegetation types. Different letters denote significant differences (*P* < 0.05).

The PCA using surviving seedling's trait data found that the first and second principle components (PC1 and PC2) explained 38.4 and 25.3% of the variance in the data, respectively. PC1 separated seedlings from one another based on the growth‐related traits (specifically the number of leaves, diameter, height, specific root length, and specific leaf area), while PC2 separated seedlings from one another based on root and leaf starch concentrations. *B. buonopozense* seedlings separated from the other species on the PC2 axis (Fig. 3). A MANOVA test showed that the separation of seedlings along PC1 was significantly influenced by vegetation type (*F* = 26.2, *P* < 0.0001) and by the interaction between vegetation type and species (*F* = 2.6, *P* = 0.02), while on PC2, it was significantly influenced by vegetation type (*F* = 34.0, *P* < 0.0001), species (*F* = 123.2, *P* < 0.0001), and the interaction between vegetation type and species (*F* = 5.1, *P* = 0.0002). Tukey tests showed that seedlings grown in the mixed vegetation type were significantly different on the PC1 axis to those grown in either forest or savanna, which were not significantly different to each other, and that seedlings from all three vegetation types were significantly different from one another on the PC2 axis (Fig. 4). Tukey tests showed that, in addition to some differences in PC1 scores between species, *T. scleroxylon* showed significant differences in PC1 scores between seedlings grown in the mixed vegetation type versus seedlings of the same species grown in the other two vegetation types (*P* < 0.0001). Similarly, *B. buonopozense* PC2 scores were near significantly different between seedlings grown in the savanna and those grown in the forest vegetation type (*P* = 0.08), while PC2 scores of *T. superba* were mostly significantly different between seedlings grown in the savanna and those grown in the other two vegetation types (savanna vs. mixed *P* = 0.003, savanna vs. forest *P* = 0.09).

Interspecies analysis showed that all four of the surviving species had significantly different root starch concentrations from one another, with *B. buonopozense* having the highest, followed by *T. scleroxylon* and then *T. superba*, with *K. ivorensis* having the lowest (Table 3). *B. buonopozense* also had the highest total amount of starch per seedling of all the species (Table 3). *B. buonopozense* showed resprouting behavior (data not shown) as well as the highest percentage of surviving seedlings (22.4%, Table 2). In contrast, *T. superba* seedlings were significantly taller and had more leaves than the other species, with the second highest total root starch per seedling (Table 3). *K. ivorensis* had significantly lower root mass and root starch concentration than the other species, as well as lower total root starch per seedling. *K. ivorensis* also had a significantly higher specific root length than the other species, which was the result of shorter roots that had a lower dry mass than the other species (Table 3).

**Table 3:** Median and interquartile range (25–75) of the variables measured in all seedlings after harvest

	BB	KI	TS	WW
Height (mm)	150^B^ (70–210)	110^B^ (90–128)	282^A^ (140–570)	150^B^ (80–365)
Diameter (mm)	4.4^A^ (3.7–6.1)	3.4^B^ (2.2–4.4)	4.6^A^ (4.4–6.2)	4.4^AB^ (2.2–6.6)
Number of leaves	5^C^ (4–7)	5^C^ (4–6)	16^A^ (11–25)	8^B^ (5–17)
Leaf dry mass (g)	0.17^A^ (0.07–0.37)	0.08^A^ (0.05–0.5)	0.73^A^ (0.10–2.00)	0.30^A^ (0.03–1.30)
Stem dry mass (g)	0.26^B^ (0.05–0.46)	0.13^B^ (0.08–0.29)	0.65^AB^ (0.23–2.11)	0.32^A^ (0.05–1.47)
Root dry mass (g)	0.77^A^ (0.46–1.20)	0.36^B^ (0.28–0.54)	1.04^A^ (0.60–1.80)	0.63^A^ (0.37–1.12)
Specific root length (mm/g)	242.54^B^ (184.17–367.54)	473.6^A^ (373.34–532.68)	235.24^B^ (166.41–325.44)	261.06^B^ (177.36–405.41)
Root:shoot (g)	1.43^A^ (0.84–3.67)	1.63^AB^ (0.42–3.93)	0.63^B^ (0.32–1.71)	0.82^AB^ (0.41–4.40)
Total root starch (mg/seedling)	51.4^A^ (27.8–77.0)	4.3^C^ (3.7–6.0)	22.0^B^ (14.6–35.9)	18.4^B^ (11.7–32.6)
[Leaf starch] (mg/g)	95.4^A^ (95.4–107.3)	16.0^C^ (5.35–22.95)	17.1^B^ (17.1–46.4)	20.4^B^ (17.6–33.8)
[Root starch] (mg/g)	56.4^A^ (56.4–63.5)	10.7^D^ (8.05–11.0)	16.5^C^ (16.5–18.6)	30.5^B^ (24.8–30.7)
[Stem starch] (mg/g)	38.8^A^ (38.8–49.5)	29.5^A^ (25.6–58.6)	20.0^B^ (14.7–20.0)	18.6^B^ (12.0–30.1)
Specific leaf area (cm^2^/g)	255.46^A^ (202.93–298.48)	223.78^A^ (200.89–247.36)	252.96^A^ (126.90–305.13)	322.62^A^ (84.66–363.42)
*n*	43	9	14	25

Species: BB = *B. buonopozense,* KI = *K. ivorensis*, ND = *N. diderrichii*, TS = *T. superba*, and WW = *T. scleroxylon*. Letters next to the median value indicate that species are significantly different (*P* < 0.05) from each other for that variable according to a nonparametric Kruskal–Wallis test. *n* is the number of surviving seedlings per species at the time of harvesting.

## Discussion

This study investigated survival of five light‐demanding tropical African forest tree seedlings across a forest–savanna transition zone, specifically examining the environmental factors and seedling traits that may be affecting seedling survival patterns during this establishment phase. Seedlings showed differential survival patterns between vegetation types, and these survival patterns were found to be associated with distinct trait features. Seedling survival was lowest in the forest vegetation type, potentially due to the limited availability of water (during dry period) and light. We also found that seedlings that were larger before fire were more likely to survive, regardless of burning.

### Seedling survival differs between vegetation types, and both fire and nonfire factors, such as light and drought, are important

Fire was shown to have a significant effect on seedling survival, which is in itself not surprising, but it does confirm that fire can be used as a management tool for controlling forest encroachment into savannas. However, in addition to fire, nonfire factors were also important in determining seedling survival. Before the fire during seedling establishment, there was a notable decrease in the number of surviving seedlings as time progressed, and since this decrease occurred before fire, we assume it to be attributable to nonfire factors such as canopy cover and drought. Seedling survival was lowest in the forest vegetation type, which had the lowest canopy openness, and therefore, less light in this environment compared with the other vegetation types with more light. It should be noted, however, that this study did not explicitly test the effects of light on seedling survival.

Cumulative rainfall also affected seedling survival, with increased drought (greater MCWD) co‐occurring with increased seedling mortality. Drought is not an uncommon challenge for savanna tree species, and savanna seedlings tend to have a deeper taproot that develops quickly at a young age to avoid physiological death by providing access to water at greater profile depth (Rizzini 1965; Moreira 1992; Oliveira and Silva 1993; Hoffmann 2000). Forest seedlings, however, do not have this ability and die more easily than savanna seedlings when both are grown in a savanna environment, which has been attributed to forest seedlings coping less well with drought stress (Hoffmann et al. 2004).

Drought and light availability can also work together to affect seedling survival rates. Light is often a limiting resource in forest environments and one of the primary factors reducing seedling growth in the forest understory as well as making forest seedlings more susceptible to drought (Veenendaal et al. 1996a, 1996b). The effects of drought can be exacerbated by a lack of light which can lead to increased forest seedling mortality under closed canopies in the dry season, particularly in light‐demanding species (Veenendaal et al. 1996b), as was observed in this study, where mortality was highest in the forest vegetation type for all species. More light functions to increase seedling survival by increasing nonstructural carbohydrate concentrations in tropical tree seedlings (O'Brien et al. 2014), increasing the seedlings' ability to survive drought (O'Brien et al. 2014) and fire (Miyanishi and Kellman 1986; Chapin et al. 1990; Bowen and Pate 1993; Iwasa and Kubo 1997; Canadell and Lopez‐Soria 1998; Hoffmann et al. 2003; Olano et al. 2006). This was seen in this study where the savanna environment, with its increased light, had the highest number of surviving seedlings at the end of the study period. However, other studies elsewhere show the opposite trend, with forest seedlings performing better in forest than in savanna environment (e.g., Bowman and Panton 1993; Hoffmann 1996; Hoffmann et al. 2004), suggesting that forest seedling survival may strongly depend on the local conditions.

Our results showed significantly higher herbaceous biomass in the savanna environments suggesting that grasses did not outcompete seedlings at the establishment phase (Table S1). The initial grass‐clipping treatment likely had little effect on grasses' competitive ability as grasses rapidly resprouted, and belowground competition for resources would have occurred throughout. Other studies have shown that while grasses may reduce emergence, growth, and survival of woody seedlings, the competitive reduction may not be large enough to cause high mortality or complete exclusion (Scholes and Archer 1997). It is also unlikely that litterfall played a role in seedling survival as the savanna environment presented the lowest litterfall (Table S1).

### Larger seedlings survived better independent of whether or not they were burnt

Seedlings that fell into a greater prefire height and diameter size class showed an increase in survival compared to those that fell into a smaller height and diameter class. This pattern was observed in burnt and unburnt seedlings, indicating that being large confers a survival advantage to seedlings, but not necessarily just because they are able to better cope with fire. This may also be due to the increased ability of larger seedlings to resist the negative effects of drought (Veenendaal et al. 1996b); however, this was not explicitly tested in our study.

These findings can be interpreted in the context of the fire resistance threshold hypothesis. The “fire resistance” threshold occurs when individual trees grow big and tall enough (usually above the grass height) to avoid top kill by fire (Higgins et al. 2000; Bond and Midgley 2001; Hoffmann et al. 2012). The time taken for tree saplings to recruit to adult tree size and cross the fire resistance threshold varies, but in arid African savanna trees, it has been estimated to be a minimum of 6 years (Wakeling et al. 2011), 8 years for neotropical savanna species, and 14 years for neotropical forest species (Hoffmann et al. 2012). There is, to the authors' knowledge, no estimate for the minimum time Afrotropical forest trees take to cross the fire resistance threshold in a mesic environment. The findings of this study are therefore important to the fire resistance threshold hypothesis as they present some evidence that the hypothesis can be applied to Afrotropical forest trees as young as 14 months. The seedlings in our study showed increased survival with increased prefire height, and while this increased survival was not explicitly linked to increased fire resistance, they do not disprove the idea that increased height confers both fire‐ and nonfire‐related survival advantages to Afrotropical forest seedlings.

It should be noted that this study may slightly overestimate seedling survival rates as the removal of herbaceous biomass when planting the seedlings would have decreased fire intensity and therefore helped seedling establish better.

### Differential survival across vegetation types was associated with distinct trait features

Results from the PCA showed that seedlings surviving in the different vegetation types had different starch (PC2)‐ and growth (PC1)‐related traits to one another. Seedling survival in savanna was strongly associated with higher leaf and root starch concentrations, traits that promote effective recovery from fire. Species that survived well in mixed environments did so based on growth and resource‐capture or competition traits, such as height, diameter, and the number of leaves. Similarly, species that survived in forest also did so based on growth and resource‐capture traits; however, these were more biomass based, such as specific root length, specific leaf area, and root‐to‐shoot ratio. These results indicate that fire mostly limits survival in savanna, where light is not limiting, while competition for light limits survival in mixed and forest environments.

In addition to this, there was also plasticity in traits on an intraspecific level, with seedlings of the same species showing significantly different traits depending on which vegetation type they were grown in. *B. buonopozense,* for example, had significantly different PC2 scores (starch traits) depending on whether it grew in savanna or forest, with similar patterns being seen in *T. superba*, whose PC2 scores were significantly different in the savanna than in the other two vegetation types. This again implies that different environmental conditions, for example, different light availabilities, lead to differential seedling success across vegetation types, even within the same species. It also supports the idea that seedling survival rates are not entirely species driven and that there are winners and losers on an individual level, with certain individuals of a species doing better than others based on their specific growing conditions. Similar results were seen in savanna tree seedlings, where certain individuals of the same species did better than others and were thus able to more effectively survive fire (Wakeling et al. 2011).

As fire is a common occurrence in savannas worldwide, many savanna trees show an evidence of allocation trade‐offs, where the ability to recover from or resist fire is traded off against the ability to grow large fast and escape fire (tall enough to keep the canopy out of flames and wide enough to have thick enough bark to prevent fire damage). For example, Brazilian savanna tree species tend to have a carbon‐expensive life history strategy, investing heavily in root carbohydrates (Hoffmann et al. 2004), root biomass (Hoffmann and Franco 2003; Tomlinson et al. 2012), and bark (Hoffmann et al. 2012), enabling them to effectively resprout after top kill by fire and quickly regrow their lost photosynthetic material (Hoffmann 2000). By contrast, Brazilian gallery forest tree species tend to have higher growth rates, but up to threefold thinner bark than savanna species, meaning that they reach fire escape height faster than savanna trees (Rossatto et al. 2009; Geiger et al. 2011; Hoffmann et al. 2012). However, this competitive advantage may be offset by their reduced fire resistance as a result of their thinner bark (Hoffmann et al. 2009). Savanna tree species in fire‐prone environments generally favor fire recovery and resistance allocation patterns (Tomlinson et al. 2012), while forest tree species living in lower light, competitive environments generally favor allocation patterns that increase shade tolerance such as fast growth (Veenendaal et al. 1996a). However, because trade‐offs can reflect adaptive traits (Tomlinson et al. 2012), forest species may be able to expand their range into savannas if they exhibit allocation patterns that favor recovery from fire rather than allocation patterns favoring fast growth.

*Bombax buonopozense* showed exactly this, it was the forest species with the highest root starch and leaf starch concentrations, and highest total root starch per seedlings, but did not exhibit greater growth and resource‐capture traits, such as height, stem diameter, or the number of leaves. This species did not survive well in the forest vegetation type, likely due to light limitation, while it had the highest survival rates in the savanna, where light allowed it to build plenty of starch reserves. Starch accumulation is a trait that likely allowed it to survive and recover effectively from fire (Miyanishi and Kellman 1986; Chapin et al. 1990; Bowen and Pate 1993; Iwasa and Kubo 1997; Canadell and Lopez‐Soria 1998; Hoffmann et al. 2003; Olano et al. 2006), and it also would have contributed to the increased prefire survival rate of this species by increasing its ability to survive drought (O'Brien et al. 2014). *B. buonopozense* preferentially allocates to fire recovery traits, similar to savanna species, which fits well with its observed natural distribution in West Africa, which includes forest–savanna transitions (Clayton 1961), and suggests that this pioneer species would be the most likely of the study species to be contributed to forest encroachment. This supports earlier work carried out by Geiger et al. (2011), who found that forest expansion into savanna is driven by a few forest and transition zone tree species.

## Conclusion

This study provides new and valuable insights into how Afrotropical forest tree seedlings survive through fire and drought during their early life stage demographic bottleneck and how these pioneer species could promote the encroachment of forest in savannas. Our key findings included showing that vegetation type affects how well seedlings survive. Seedlings that survived better in mixed environments invested more in growth‐related traits, while seedlings that survived better in savanna environments invested more in starch‐related traits. Survival of the pioneer forest tree species was the lowest in forest environments, likely as a result of decreased access to light. Larger seedlings survive both fire and drought better than smaller seedlings. *B. buonopozense* was the species with the highest survival rate of all the species, and it survived best in the savanna environment. This was likely due to the increased access to light in the open‐canopied savanna, which enabled these seedlings to accumulate more starch and therefore survive better through drought and fire. Due to its ability to survive better in the savanna environment than any other species, we recommend to managers that *B. buonopozense* would be a good indicator species of forest encroachment into savanna. We also recommend that fire would be a good management tool to prevent this as fire had a significant effect on seedling survival.

## Conflict of Interest

None declared.

## Supporting information


**Figure S1.** Calibration equation of the disc pasture meter.

**Table S1.** Average (±standard error) herbaceous biomass and litterfall per vegetation type before the fire (January 2014).
**Table S2**. Mantel‐Cox log‐rank test results for survival before fire.
