# Supplementary material for: Winners and losers: tropical forest tree seedling survival across a West African forest–savanna transition
Source: Ecol Evol. 2016 Apr 18;6(10):3417–29. doi: 10.1002/ece3.2133 (PMC4840012; doi:10.1002/ece3.2133)
Supplement: Supplementary file 2 — Table S1. Average (±standard error) herbaceous biomass and litterfall per vegetation type before the fire (January 2014). Table S2. Mantel‐Cox log‐rank test results for survival before fire. [file ECE3-6-3417-s002.docx]

**SUPPLEMENTARY MATERIAL**

**Table S1:** Average (± standard error) herbaceous biomass and litterfall per vegetation type before the fire (January 2014). Vegetation types are: forest=FOR, mixed-MXD, savanna=SAV. Superscript letters indicate significant differences (canopy openness and herbaceous biomass Kruskal-Wallis test, litterfall one-Way ANOVA) between vegetation types at p < 0.05.

|  | FOR | MXD | SAV |
| --- | --- | --- | --- |
| Canopy openness | 19.2 ± 0.9^c^ | 25.6 ± 0.6^b^ | 54.4 ±3.0^a^ |
| Herbaceous biomass (kg/ha) | 476 ± 29^a^ | 535 ± 40^a^ | 3646 ± 572^b^ |
| Litterfall (kg/ha) | 3917 ± 348^a^ | 3848 ± 785^a^ | 2139 ± 437^b^ |

**Table S2**: Mantel-Cox log-rank test results for survival before fire. Species names are: BB=*B.buonopozense,* KI=*K.ivorensis*, ND=*N.diderrichii*, TS=*T.superba* and WW=*T.scleroxylon.*

|  | | | | | | | | | | | |
| --- | --- | --- | --- | --- | --- | --- | --- | --- | --- | --- | --- |
|  | species | BB | | KI | | ND | | TS | | WW | |
|  |  | χ^2^ | Sig. | χ^2^ | Sig. | χ^2^ | Sig. | χ^2^ | Sig. | χ^2^ | Sig. |
| Log Rank (Mantel-Cox) | BB |  |  | 6.827 | .009 | 36.863 | .000 | 9.089 | .003 | .593 | .441 |
|  | KI | 6.827 | .009 |  |  | 12.382 | .000 | .165 | .685 | 3.392 | .066 |
|  | ND | 36.863 | .000 | 12.382 | .000 |  |  | 9.724 | .002 | 28.268 | .000 |
|  | TS | 9.089 | .003 | .165 | .685 | 9.724 | .002 |  |  | 5.036 | .025 |
|  | WW | .593 | .441 | 3.392 | .066 | 28.268 | .000 | 5.036 | .025 |  |  |

**Figure S1.** Calibration equation of the disc pasture meter. At each vegetation type, we randomly selected five 50 x 50 cm plots in which we took four disc pasture meter readings and clipped all the biomass. Biomass was calculated by oven-dried at 72°C until constant weight. Biomass and the disc pasture meter readings were fitted to a linear model.
